# Multidisciplinary approach of liver metastases from colorectal cancer

**DOI:** 10.1002/ags3.12227

**Published:** 2019-01-14

**Authors:** René Adam, Yuki Kitano

**Affiliations:** ^1^ AP‐HP Paul Brousse Hospital, Hepato‐Biliary Center Paris Sud University Inserm U 935 Villejuif France; ^2^ Department of Gastroenterological Surgery Graduate School of Medical Sciences Kumamoto University Kumamoto Japan

**Keywords:** colorectal cancer liver metastases, multidisciplinary approach, OncoSurge approach, unresectable colorectal cancer liver metastases

## Abstract

Colorectal cancer liver metastases (CRLM) represent most of the causes of death in patients with colorectal cancer. Surgical resection is the only treatment that can provide the possibility of prolonged survival, or even cure, for patients with CRLM. Over the last few decades, survival of these patients has improved dramatically thanks to more effective chemotherapy, extension of surgical indications, and development of new surgical procedures. In particular, patients with initially unresectable CRLM can achieve downsizing of the tumor by using various chemotherapies and the tumor can become resectable. It has been shown that such patients have a 33% 5‐year survival and a 23% 10‐year survival rate after surgery, which is a little bit lower than that of patents with resectable CRLM but significantly higher than patients without surgery. However, a decision‐making strategy for patients with CRLM is difficult because there is a wide variety of treatments and no definitive consensus. As an example, much variation among institutions exists on the resectability rate in patients with unresectable CRLM. Also, it is recommended that all patients with CRLM be managed by a multidisciplinary approach (MDA) to select the best strategy. In the future, new treatment procedures (e.g. immune checkpoint blockade, liver transplantation) may contribute to improve prognosis; hence, the necessity for MDA for the treatment of CRLM will further increase.

## 1 INTRODUCTION

Worldwide, colorectal cancer (CRC) is responsible for 1 400 000 new cases and more than 600 000 deaths each year, two‐thirds of which are related to liver metastases.^1^ It is a major public health problem. Moreover, in Asia, including Japan, China, South Korea, and Singapore, the incidence of CRC has increased two‐ to fourfold in the past two decades.^2^ In patients with CRC, the liver is the most common site of metastases and approximately half of patients develop liver metastases during the course of their disease.^3^ Hepatic resection is the only treatment that can provide the possibility of prolonged survival or even cure for patients with colorectal liver metastases (CRLM). It is associated with 5‐year survival rates ranging from 16% to 71%.^4^ Moreover, although approximately 80% of patients with CRLM are unfortunately found to be unresectable at the time of diagnosis, recent innovations in the treatment of CRLM have enabled hepatic resection for such patients, and their 5‐year survival rate has reached 33% to 50%.^5^, ^6^, ^7^ Consequently, the treatment strategy for CRLM, either initially resectable or unresectable, should be directed toward their potential resectability.

In recent decades, much effort has been made to increase resectability for patients with unresectable CRLM, and various strategies have been established to improve their prognosis.^4^, ^8^ First, advancement of systemic chemotherapy with or without targeted therapies has enabled surgical treatment for even initially unresectable CRLM by downsizing the tumors, by the so‐called “OncoSurge approach”.^4^, ^9^ Currently, this strategy has shown clinical benefit in many studies and a recent systematic review reported that the objective response rate and the R0 resection rate were 64% and 87%, respectively.^7^ Second, a shift of surgical indications for CRLM from old criteria has enlarged the population of resectable CRLM.^4^, ^10^, ^11^, ^12^ Innovations in surgical techniques and perioperative management have pushed the boundaries of resectability, and many published studies have shown the efficacy and safety of these shifts.^4^, ^13^, ^14^, ^15^, ^16^, ^17^, ^18^ Third, the development of surgical procedures such as portal vein embolization (PVE),^19^, ^20^ radiofrequency ablation (RFA) combined with hepatectomy,^21^ two‐stage hepatectomy (TSH),^22^, ^23^, ^24^ and associating liver partition and portal vein ligation for staged hepatectomy (ALPPS)^25^, ^26^ have expanded the indications of surgery for patients with unresectable CRLM with a survival benefit to selected patients.^4^, ^27^

Management of CRLM is difficult in the absence of data from randomized controlled trials (RCT) to guide decisions and because of the wide variety of factors influencing the strategy (e.g. initial resectability, synchronicity of CRLM, timing of surgery, role of laparoscopic surgery, type of chemotherapy regimen, and pre/postoperative management).^8^ Physicians must personalize treatment for each patient. To achieve this objective, multidisciplinary approach (MDA) has increasingly been implemented for cancer care services.^28^, ^29^ For the improvement of patients’ prognosis, the treatment strategy for CRLM should be directed toward resectability, and it is recommended that all patients with CRLM should be treated by specialized multidisciplinary teams (MDT) individually to decide the best strategy.^4^, ^8^, ^30^ In this article, we address the importance of MDA for the management of CRLM and topics that are currently under debate to personalize the treatment strategy for each patient in relation to his/her disease.

## 2 MULTIDISCIPLINARY APPROACH FOR CRLM

### 2.1 Chemotherapy to achieve resectability

**Figure 1:**
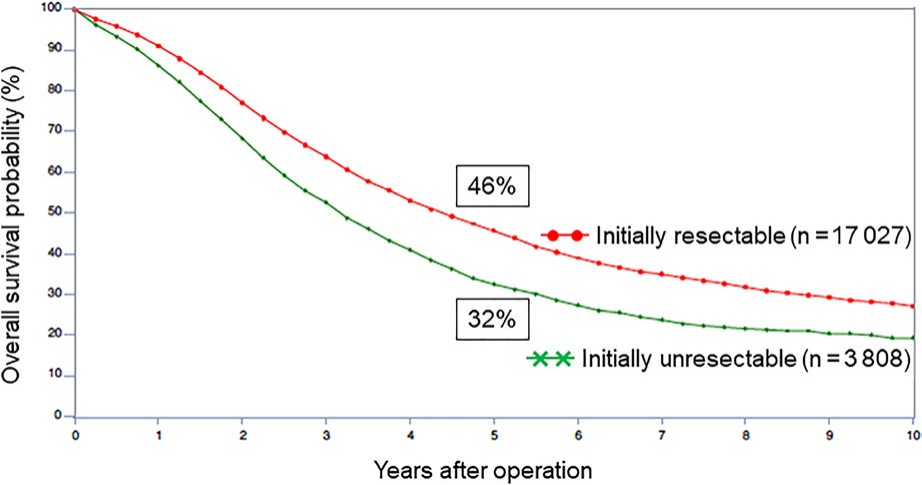
Overall survival probability after hepatic resection of initially resectable vs unresectable colorectal cancer liver metastases

The majority of patients with CRLM are initially unresectable, and they must be treated by chemotherapy to achieve resectability as their prognosis is obviously much better if metastases can be removed surgically than if they cannot be resected.^5^, ^6^, ^7^ The LiverMetSurvey International Registry now involving over 25 000 patients from 326 centers in 71 countries has shown that the 5‐year survival rate was 32% for approximately 4000 initially unresectable patients that became resectable thanks to efficient chemotherapy (Figure 1).

Which are the best conditions for an OncoSurge approach? The first is optimal first‐line chemotherapy because there is a strong correlation between the resection rate and the response rate to chemotherapy.^31^, ^32^ Now, some image assessments (e.g. early tumor shrinkage, depth of response, and metabolic response) evaluating response to preoperative chemotherapy are used as a surrogate marker to predict long‐term outcome.^33^, ^34^ In the last two decades, chemotherapy has made tremendous progress. Before 1990, 5‐fluorouracil (5‐FU) was the only chemotherapeutic option for metastatic CRC; then in 1990‐2000, oxaliplatin and irinotecan became available and doublet cytotoxic regimens became standard therapy as FOLFOX (5‐FU, leucovorin, and oxaliplatin) and FOLFIRI (5‐FU, leucovorin, and irinotecan).^32^ More recently, the use of triplet cytotoxic combinations with 5‐FU plus both oxaliplatin and irinotecan (FOLFOXIRI) has translated into higher response and resectability rates.^35^ Moreover, by adding targeted therapies, anti‐epidermal growth factor receptor (anti‐EGFR) or anti‐vascular endothelial growth factor (anti‐VEGF) antibodies to these regimens, high response rates (>50%) and long median survival (~30 months) have been achieved.^8^ Regarding the better choice of targeted therapies for optimal first‐line chemotherapy, tumor *RAS* mutational status should be taken into consideration as a pretreatment biomarker. Patients with *RAS* wild‐type disease should be treated with anti‐EGFR antibody‐containing regimen as first‐line chemotherapy followed by anti‐VEGF antibody‐containing regimen.^36^, ^37^ Meanwhile, patients with *RAS* mutant disease or unknown *RAS* status should be treated with anti‐VEGF antibody‐containing regimen.^38^ To further increase the resectability rate or treat patients who escape to conventional chemotherapy, another possibility is hepatic artery infusion (HAI). Several studies have shown that HAI could provide a better response rate, a high rate of secondary resection with downsizing in the majority of tumors, and good survival rates.^39^, ^40^ This was recently confirmed by a European RCT showing that using triplet infusion of 5‐FU, oxaliplatin, and irinotecan into the hepatic artery with systemic cetuximab in patients with RAS wild type having a median of 10 liver metastases, the primary endpoint was met with a R0‐R1 hepatectomy achieved in 30% of the patients and a high response rate, even after they have escaped one or two lines of systemic chemotherapy.^41^ Thus, HAI may be a second chance for patients with unresectable CRLM not responding to systemic chemotherapy. Furthermore, recently, the efficacy of an immune checkpoint blockade for various types of cancer has been established.^42^ In patients with metastatic CRC, the objective response rate of antiprogrammed death‐1 antibody was 40%‐69% for mismatch repair‐deficient CRC.^43^, ^44^ In future clinical practice, further investigation of immune checkpoint blockade is warranted for use in patients with CRLM.

The second condition is a short duration of first‐line chemotherapy. This means that preoperative treatment to induce resectability should be as short as possible because the more cycles we do before surgery, the more toxicity the liver may suffer, and the fewer cycles we can deliver after surgery. We know that prolonged chemotherapy causes a “blue liver” related to giving oxaliplatin or a “yellow (steatotic) liver” related to prolonged dosage of irinotecan, and these livers are exposed to a higher risk of morbidity and mortality.^45^, ^46^ In addition, because the aim of the OncoSurge approach is to achieve resectability only, not a complete response, we recommend that the optimal timing for the assessment of response to chemotherapy is every 2 months. When there is a possibility of resectability, at least four courses of chemotherapy should be given as first line, and if either progression or stable disease occurs during first‐line treatment after 4 months, second‐line treatment should be considered. Overall, a total duration of 6 months of perioperative chemotherapy is recommended.^4^, ^47^ In terms of adjuvant chemotherapy, there is no definitive consensus for adjuvant chemotherapy in patients with resection of CRLM, although some studies have shown an advantage of adjuvant chemotherapy.^4^, ^48^, ^49^ Surgery should be avoided in cases of progression as the survival benefit of patients who underwent hepatic resection during progression of disease while on chemotherapy is much more limited than that of patients with partial response or stability.^50^

Therefore, how can we treat patients escaping first‐line chemotherapy? From the LiverMetSurvey International Registry, results of hepatic resection for unresectable CRLM after second‐line chemotherapy are comparable to that after first‐line chemotherapy.^51^ Consequently, physicians should continue to seek the opportunity of surgical intervention, even after failure of first‐line chemotherapy.

### 2.2 Shift of the surgical indication for CRLM

**Figure 2:**
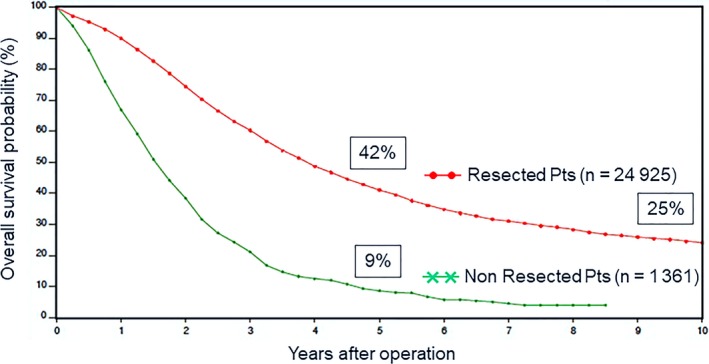
Overall survival probability of patients with colorectal cancer liver metastases resected vs unresected

Hepatic resection is the only treatment that can provide the possibility of prolonged survival or even cure for patients with CRLM.^4^, ^6^ The LiverMetSurvey International Registry shows that in patients with CRLM who underwent hepatic resection, the 5‐ and 10‐year survival rates are 42% and 25%, respectively, and that of operated but non‐resected patients is only 9% (Figure 2). Although resection is the only means to prolong survival, a minority of patients with CRLM are resectable at the time of diagnosis.^5^ The recent progression of surgery for CRLM is a shift of surgical indications from old criteria that were very strict (e.g. fewer than three metastases, less than 5‐cm maximal size, negative resection margin, and low preoperative carcinoembryonic antigen [CEA] levels etc.).^4^, ^10^, ^11^, ^12^ Resectability and curability are dependent on multiple factors, including number and location of metastases, volume of the future liver remnant, presence of extrahepatic disease, and the patient's general condition. The most important shift in the indications is the number of metastases. In the past, the presence of more than three CRLM was considered a contraindication for resection.^10^ Although innovations in surgical techniques and perioperative management have increased the chance for surgery in patients with unresectable CRLM, the oncological dogma of “no more than three CRLM” has been progressively challenged.^4^ Now, resection of multiple bilobar hepatic metastases has shown survival benefits and even patients with more than 10 metastases may have a 30% 5‐year survival after resection.^13^, ^14^ Another shift is surgical resection margin. Of course, the gold standard for the surgical management of CRLM is complete resection with histologically negative margins (R0 resection).^52^ Several studies have shown that the so‐called R1 resection (tumor‐free margin <1 mm) is associated with worse overall survival (OS) than R0 resection (tumor‐free margin ≥1 mm).^53^ However, it is sometimes impossible to achieve adequate surgical margins as a result of contact of the tumor with vascular structures and, by necessity, R1 resection could be worthwhile. In the current era of effective chemotherapy, surgical margin status did not impact survival status in patients who received perioperative chemotherapy, especially for patients with a good response.^15^, ^17^ The last point of shift is surgery for elderly patients with CRLM. Even considering patients more than 80 years old, long‐term survival is valuable.^16^, ^18^ Consequently, many factors that previously contraindicated surgery have changed and the criteria for surgical indications for CRLM are permanently evolving.

### 2.3 Development of new surgical procedures

In the past, patients with unresectable disease were treated with palliative chemotherapy.^4^ In recent decades, many efforts have been made to increase resectability for patients with unresectable CRLM, and various strategies have been established.^4^, ^8^, ^27^ PVE was developed for patients with extended hepatectomy to induce ipsilateral atrophy and contralateral compensatory hypertrophy of the remnant liver, thereby preventing severe postoperative liver failure,^19^ and PVE increased the resectability rate of initially unresectable CRLM.^20^ Likewise, RFA combined with hepatectomy has been shown to be safe and feasible in patients with unresectable CRLM.^21^ However, for patients with extensive bilateral multinodular CRLM, a single hepatectomy even with such specific procedures as PVE or RFA could be insufficient to remove all of the tumors. In 2000, we reported the concept of TSH, based on two sequential procedures to remove multiple bilateral tumors that are impossible to remove by a single hepatectomy. The procedure uses the liver regeneration obtained after the first procedure.^22^ During the next decade, this procedure has evolved in combination with PVE and effective chemotherapy and has been adopted by many specialized centers worldwide with promising short‐ and long‐term outcomes.^23^, ^24^, ^54^ However, its major drawback is the dropout risk as a result of disease progression and insufficient hypertrophy between the two stages, making resection impossible in 25%‐38% of patients prepared to undergo TSH.^24^, ^55^, ^56^, ^57^ To overcome this dropout risk, a German group has demonstrated an alternative treatment, so‐called ALPPS.^25^, ^26^ They have added TSH to a splitting of the liver in a plan of future hepatectomy and demonstrated a volume increase of 74% of the remnant liver in a period of only 9 days, allowing TSH to be done even during the same hospitalization of the patient.^26^ Although the advantage of ALPPS is, of course, its high feasibility, its increased frequency of severe complications and perioperative mortality are worrisome problems, and its long‐term outcome is also controversial. In the initial study, 68% of the patients experienced complications and the surgical mortality rate was 12%. Recently, from a North European group, RCT evaluating the early outcome between ALPPS and TSH showed that morbidity and mortality rates are comparable, as the feasibility of ALPPS is also higher.^57^ However, long‐term outcomes still remain to be elucidated. In our initial experience, the survival rate seemed to be lower after ALPPS as compared to TSH.^26^, ^58^ In order to elucidate the role of ALPPS regarding oncological outcomes, further studies are hopefully expected. Despite these advances in surgical procedures, many patients with CRLM are still regarded as unresectable. For such patients, today, we may reconsider the possibility of liver transplantation (LT). In the past, LT for patients with CRLM was an absolute contraindication because of organ shortage and the low long‐term survival. One‐ and 5‐year survival following LT for CRLM carried out before 1995 was 62% and 18%, respectively, and perioperative mortality after LT was approximately 30%.^59^ However, nowadays, much has changed: better expertise in the management of LT, better knowledge of the biology of metastatic disease, better imaging techniques for proper patient selection, and more effective chemotherapy with immunosuppressive effects. Thanks to such dramatic progress, we have suggested revisiting the indications for LT as a treatment option for CRLM.^60^, ^61^ Following our proposal, a group in Oslo, Norway showed that the 5‐year survival rate of patients who underwent LT for unresectable CRLM was 60%, and LT resulted in a marked increased OS in such patients, compared with chemotherapy alone.^62^, ^63^ Today, a randomized multicentric trial comparing LT with chemotherapy is ongoing with 17 French and eight European centers to elucidate the role of LT as the strategy for unresectable CRLM.

### 2.4 Multidisciplinary approach for CRLM

The strategies discussed herein are only possible with discussion of MDA and this approach is increasingly favored for cancer care.^28^ From a first prospective study to assess the impact of MDT in various gastrointestinal cancers, changes of treatment recommendations occurred in more than one‐third of patients after discussion of MDT.^64^ Also, for the treatment of patients with CRLM, it has been proposed that all patients with CRLM be managed by specialized hepatobiliary MDT to select the best strategy.^4^, ^8^, ^30^, ^47^ MDA is a patient‐centered approach routinely carried out by such experts as medical oncologists, surgeons, radiologists, and pathologists, and the team works together to choose the appropriate treatment, with feedback to patients and physicians.^4^, ^28^, ^29^ Hence, the key to MDA is collaboration between specialists. Advantages of MDA include no dogmatic decisions, quick decision strategy, dynamic re‐evaluation of the patient with good treatment at good timing, and synergy in the efficiency of all treatments. To improve patients’ prognosis by MDA, the medical oncologist requires the surgeon for resectability decision‐making and timing of surgery. Meanwhile, the surgeon needs the medical oncologist to make unresectable patients resectable, to control the disease before surgery, and to prevent recurrence after surgery. Another key of MDA is the necessity for expert decision‐making on treatment strategies for CRLM. It was reported that almost two‐thirds of patients with CRLM deemed unresectable by non‐specialists were considered potentially resectable by experts in liver surgery.^65^ This means that management of patients with CRLM without the involvement of a liver surgery specialist can deny patients potentially curative treatment. In connection with this, a population‐based study of hepatic resection for CRLM across England showed that the rate of hepatic resection for CRLM varied very significantly across hospitals.^66^ Also, in the FIRE‐3 trial, the resectability rate among hospitals varied from 25% in university hospitals to 16% in non‐university hospitals, and to only 10% in medical practice oncology or private institutions.^67^ These variations relied on the absence of expertise in some centers. Consequently, expertise is very important to try to expand the surgical indications and to select the best strategy for patients with CRLM.

## 3 CONCLUSIONS

The aim of treatments for patients with CRLM is to achieve liver resection, which provides the best long‐term patient outcome. Over the last two decades, the advent of more effective chemotherapy as well as the development of new surgical procedures has dramatically improved the prognosis. In the future, new treatment procedures (e.g. immune checkpoint blockade, LT) are still likely to contribute to an improved outcome. We recommend that all patients with CRLM be managed by specialized hepatobiliary MDT to select the best strategy. All specialists must work together with a common aim for the patients: the possibility of cure and, if not, that of prolonged survival.

## DISCLOSURE

Authors declare no conflicts of interest for this article.

## References

[1] Ferlay J , Soerjomataram I , Dikshit R , et al. Cancer incidence and mortality worldwide: sources, methods and major patterns in GLOBOCAN 2012. Int J Cancer. 2015;136:E359–86. 25220842 10.1002/ijc.29210

[2] Sung JJ , Lau JY , Goh KL , Leung WK , Asia Pacific Working Group on Colorectal C . Increasing incidence of colorectal cancer in Asia: implications for screening. Lancet Oncol. 2005;6:871–6. 16257795 10.1016/S1470-2045(05)70422-8

[3] Manfredi S , Lepage C , Hatem C , Coatmeur O , Faivre J , Bouvier AM . Epidemiology and management of liver metastases from colorectal cancer. Ann Surg. 2006;244:254–9. 16858188 10.1097/01.sla.0000217629.94941.cf PMC1602156

[4] Adam R , De Gramont A , Figueras J , et al. The oncosurgery approach to managing liver metastases from colorectal cancer: a multidisciplinary international consensus. Oncologist. 2012;17:1225–39. 22962059 10.1634/theoncologist.2012-0121 PMC3481888

[5] Adam R , Delvart V , Pascal G , et al. Rescue surgery for unresectable colorectal liver metastases downstaged by chemotherapy: a model to predict long‐term survival. Ann Surg. 2004;240:644–57; discussion 57‐8. 15383792 10.1097/01.sla.0000141198.92114.f6 PMC1356466

[6] Adam R , Wicherts DA , de Haas RJ , et al. Patients with initially unresectable colorectal liver metastases: is there a possibility of cure? J Clin Oncol. 2009;27:1829–35. 19273699 10.1200/JCO.2008.19.9273

[7] Lam VW , Spiro C , Laurence JM , et al. A systematic review of clinical response and survival outcomes of downsizing systemic chemotherapy and rescue liver surgery in patients with initially unresectable colorectal liver metastases. Ann Surg Oncol. 2012;19:1292–301. 21922338 10.1245/s10434-011-2061-0

[8] Adam R , de Gramont A , Figueras J , et al. Managing synchronous liver metastases from colorectal cancer: a multidisciplinary international consensus. Cancer Treat Rev. 2015;41:729–41. 26417845 10.1016/j.ctrv.2015.06.006

[9] Poston GJ , Adam R , Alberts S , et al. OncoSurge: a strategy for improving resectability with curative intent in metastatic colorectal cancer. J Clin Oncol. 2005;23:7125–34. 16192596 10.1200/JCO.2005.08.722

[10] Ekberg H , Tranberg KG , Andersson R , et al. Determinants of survival in liver resection for colorectal secondaries. Br J Surg. 1986;73:727–31. 3756436 10.1002/bjs.1800730917

[11] Fong Y , Fortner J , Sun RL , Brennan MF , Blumgart LH . Clinical score for predicting recurrence after hepatic resection for metastatic colorectal cancer: analysis of 1001 consecutive cases. Ann Surg. 1999;230:309–18; discussion 18‐21. 10493478 10.1097/00000658-199909000-00004 PMC1420876

[12] Margonis GA , Buettner S , Andreatos N , et al. Prognostic factors change over time after hepatectomy for colorectal liver metastases: a multi‐institutional, international analysis of 1099 patients. Ann Surg. 2018. [Epub ahead of print]. 10.1097/SLA.0000000000002664 31082912

[13] Allard MA , Adam R , Giuliante F , et al. Long‐term outcomes of patients with 10 or more colorectal liver metastases. Br J Cancer. 2017;117:604–11. 28728167 10.1038/bjc.2017.218 PMC5572175

[14] Bolton JS , Fuhrman GM . Survival after resection of multiple bilobar hepatic metastases from colorectal carcinoma. Ann Surg. 2000;231:743–51. 10767796 10.1097/00000658-200005000-00015 PMC1421062

[15] de Haas RJ , Wicherts DA , Flores E , Azoulay D , Castaing D , Adam R . R1 resection by necessity for colorectal liver metastases: is it still a contraindication to surgery? Ann Surg. 2008;248:626–37. 18936576 10.1097/SLA.0b013e31818a07f1

[16] Adam R , Frilling A , Elias D , et al. Liver resection of colorectal metastases in elderly patients. Br J Surg. 2010;97:366–76. 20101645 10.1002/bjs.6889

[17] Hosokawa I , Allard MA , Gelli M , et al. Long‐Term Survival Benefit and Potential for Cure after R1 Resection for Colorectal Liver Metastases. Ann Surg Oncol. 2016;23:1897–905. 26822881 10.1245/s10434-015-5060-8

[18] De Blasi V , Memeo R , Adam R , et al. Major hepatectomy for colorectal liver metastases in patients aged over 80: a propensity score matching analysis. Dig Surg. 2018;35(4):333–41. 29669343 10.1159/000486522

[19] Makuuchi M , Thai BL , Takayasu K , et al. Preoperative portal embolization to increase safety of major hepatectomy for hilar bile duct carcinoma: a preliminary report. Surgery. 1990;107:521–7. 2333592

[20] Wicherts DA , de Haas RJ , Andreani P , et al. Impact of portal vein embolization on long‐term survival of patients with primarily unresectable colorectal liver metastases. Br J Surg. 2010;97:240–50. 20087967 10.1002/bjs.6756

[21] Imai K , Allard MA , Castro Benitez C , et al. Long‐term outcomes of radiofrequency ablation combined with hepatectomy compared with hepatectomy alone for colorectal liver metastases. Br J Surg. 2017;104:570–9. 28112813 10.1002/bjs.10447

[22] Adam R , Laurent A , Azoulay D , Castaing D , Bismuth H . Two‐stage hepatectomy: A planned strategy to treat irresectable liver tumors. Ann Surg. 2000;232:777–85. 11088072 10.1097/00000658-200012000-00006 PMC1421270

[23] Wicherts DA , Miller R , de Haas RJ , et al. Long‐term results of two‐stage hepatectomy for irresectable colorectal cancer liver metastases. Ann Surg. 2008;248:994–1005. 19092344 10.1097/SLA.0b013e3181907fd9

[24] Imai K , Benitez CC , Allard MA , et al. Failure to achieve a 2‐stage hepatectomy for colorectal liver metastases: how to prevent it? Ann Surg. 2015;262:772–8; discussion 778‐9. 26583665 10.1097/SLA.0000000000001449

[25] de Santibanes E , Clavien PA . Playing Play‐Doh to prevent postoperative liver failure: the “ALPPS” approach. Ann Surg. 2012;255:415–7. 22330039 10.1097/SLA.0b013e318248577d

[26] Schnitzbauer AA , Lang SA , Goessmann H , et al. Right portal vein ligation combined with in situ splitting induces rapid left lateral liver lobe hypertrophy enabling 2‐staged extended right hepatic resection in small‐for‐size settings. Ann Surg. 2012;255:405–14. 22330038 10.1097/SLA.0b013e31824856f5

[27] Torzilli G , Adam R , Vigano L , et al. Surgery of colorectal liver metastases: pushing the limits. Liver Cancer. 2016;6:80–9. 27995092 10.1159/000449495 PMC5159716

[28] Fennell ML , Das IP , Clauser S , Petrelli N , Salner A . The organization of multidisciplinary care teams: modeling internal and external influences on cancer care quality. J Natl Cancer Inst Monogr. 2010;2010:72–80. 20386055 10.1093/jncimonographs/lgq010 PMC3482953

[29] Taylor C , Munro AJ , Glynne‐Jones R , et al. Multidisciplinary team working in cancer: what is the evidence? BMJ. 2010;340:c951. 20332315 10.1136/bmj.c951

[30] Jones RP , Hamann S , Malik HZ , Fenwick SW , Poston GJ , Folprecht G . Defined criteria for resectability improves rates of secondary resection after systemic therapy for liver limited metastatic colorectal cancer. Eur J Cancer. 2014;50:1590–601. 24661798 10.1016/j.ejca.2014.02.024

[31] Folprecht G , Grothey A , Alberts S , Raab HR , Kohne CH . Neoadjuvant treatment of unresectable colorectal liver metastases: correlation between tumour response and resection rates. Ann Oncol. 2005;16:1311–9. 15870084 10.1093/annonc/mdi246

[32] Adam R , Haller DG , Poston G , et al. Toward optimized front‐line therapeutic strategies in patients with metastatic colorectal cancer–an expert review from the International Congress on Anti‐Cancer Treatment (ICACT) 2009. Ann Oncol. 2010;21:1579–84. 20219759 10.1093/annonc/mdq043

[33] Lau LF , Williams DS , Lee ST , Scott AM , Christophi C , Muralidharan V . Metabolic response to preoperative chemotherapy predicts prognosis for patients undergoing surgical resection of colorectal cancer metastatic to the liver. Ann Surg Oncol. 2014;21:2420–8. 24595797 10.1245/s10434-014-3590-0

[34] Heinemann V , Stintzing S , Modest DP , Giessen‐Jung C , Michl M , Mansmann UR . Early tumour shrinkage (ETS) and depth of response (DpR) in the treatment of patients with metastatic colorectal cancer (mCRC). Eur J Cancer. 2015;51:1927–36. 26188850 10.1016/j.ejca.2015.06.116

[35] Falcone A , Ricci S , Brunetti I , et al. Phase III trial of infusional fluorouracil, leucovorin, oxaliplatin, and irinotecan (FOLFOXIRI) compared with infusional fluorouracil, leucovorin, and irinotecan (FOLFIRI) as first‐line treatment for metastatic colorectal cancer: the Gruppo Oncologico Nord Ovest. J Clin Oncol. 2007;25:1670–6. 17470860 10.1200/JCO.2006.09.0928

[36] Schwartzberg LS , Rivera F , Karthaus M , et al. PEAK: a randomized, multicenter phase II study of panitumumab plus modified fluorouracil, leucovorin, and oxaliplatin (mFOLFOX6) or bevacizumab plus mFOLFOX6 in patients with previously untreated, unresectable, wild‐type KRAS exon 2 metastatic colorectal cancer. J Clin Oncol. 2014;32:2240–7. 24687833 10.1200/JCO.2013.53.2473

[37] Heinemann V , von Weikersthal LF , Decker T , et al. FOLFIRI plus cetuximab versus FOLFIRI plus bevacizumab as first‐line treatment for patients with metastatic colorectal cancer (FIRE‐3): a randomised, open‐label, phase 3 trial. Lancet Oncol. 2014;15:1065–75. 25088940 10.1016/S1470-2045(14)70330-4

[38] Hurwitz HI , Yi J , Ince W , Novotny WF , Rosen O . The clinical benefit of bevacizumab in metastatic colorectal cancer is independent of K‐ras mutation status: analysis of a phase III study of bevacizumab with chemotherapy in previously untreated metastatic colorectal cancer. Oncologist. 2009;14:22–8. 10.1634/theoncologist.2008-0213 19144677

[39] Kemeny N , Huang Y , Cohen AM , et al. Hepatic arterial infusion of chemotherapy after resection of hepatic metastases from colorectal cancer. N Engl J Med. 1999;341:2039–48. 10615075 10.1056/NEJM199912303412702

[40] D'Angelica MI , Correa‐Gallego C , Paty PB , et al. Phase II trial of hepatic artery infusional and systemic chemotherapy for patients with unresectable hepatic metastases from colorectal cancer: conversion to resection and long‐term outcomes. Ann Surg. 2015;261:353–60. 24646562 10.1097/SLA.0000000000000614 PMC4578807

[41] Levi FA , Boige V , Hebbar M , et al. Conversion to resection of liver metastases from colorectal cancer with hepatic artery infusion of combined chemotherapy and systemic cetuximab in multicenter trial OPTILIV. Ann Oncol. 2016;27:267–74. 26578731 10.1093/annonc/mdv548

[42] Brahmer JR , Tykodi SS , Chow LQ , et al. Safety and activity of anti‐PD‐L1 antibody in patients with advanced cancer. N Engl J Med. 2012;366:2455–65. 22658128 10.1056/NEJMoa1200694 PMC3563263

[43] Overman MJ , McDermott R , Leach JL , et al. Nivolumab in patients with metastatic DNA mismatch repair‐deficient or microsatellite instability‐high colorectal cancer (CheckMate 142): an open‐label, multicentre, phase 2 study. Lancet Oncol. 2017;18:1182–91. 28734759 10.1016/S1470-2045(17)30422-9 PMC6207072

[44] Le DT , Uram JN , Wang H , et al. PD‐1 blockade in tumors with mismatch‐repair deficiency. N Engl J Med. 2015;372:2509–20. 26028255 10.1056/NEJMoa1500596 PMC4481136

[45] Fernandez FG , Ritter J , Goodwin JW , Linehan DC , Hawkins WG , Strasberg SM . Effect of steatohepatitis associated with irinotecan or oxaliplatin pretreatment on resectability of hepatic colorectal metastases. J Am Coll Surg. 2005;200:845–53. 15922194 10.1016/j.jamcollsurg.2005.01.024

[46] Soubrane O , Brouquet A , Zalinski S , et al. Predicting high grade lesions of sinusoidal obstruction syndrome related to oxaliplatin‐based chemotherapy for colorectal liver metastases: correlation with post‐hepatectomy outcome. Ann Surg. 2010;251:454–60. 20160638 10.1097/SLA.0b013e3181c79403

[47] Nordlinger B , Vauthey JN , Poston G , Benoist S , Rougier P , Van Cutsem E . The timing of chemotherapy and surgery for the treatment of colorectal liver metastases. Clin Colorectal Cancer. 2010;9:212–8. 20920992 10.3816/CCC.2010.n.031

[48] Khoo E , O'Neill S , Brown E , Wigmore SJ , Harrison EM . Systematic review of systemic adjuvant, neoadjuvant and perioperative chemotherapy for resectable colorectal‐liver metastases. HPB (Oxford). 2016;18:485–93. 27317952 10.1016/j.hpb.2016.03.001 PMC4913134

[49] Adam R , Bhangui P , Poston G , et al. Is perioperative chemotherapy useful for solitary, metachronous, colorectal liver metastases? Ann Surg. 2010;252:774–87. 21037433 10.1097/SLA.0b013e3181fcf3e3

[50] Adam R , Pascal G , Castaing D , et al. Tumor progression while on chemotherapy: a contraindication to liver resection for multiple colorectal metastases? Ann Surg. 2004;240:1052–61; discussion 61‐4. 15570210 10.1097/01.sla.0000145964.08365.01 PMC1356520

[51] Adam R , Yi B , Innominato PF , et al. Resection of colorectal liver metastases after second‐line chemotherapy: is it worthwhile? A LiverMetSurvey analysis of 6415 patients. Eur J Cancer. 2017;78:7–15. 28407529 10.1016/j.ejca.2017.03.009

[52] Charnsangavej C , Clary B , Fong Y , Grothey A , Pawlik TM , Choti MA . Selection of patients for resection of hepatic colorectal metastases: expert consensus statement. Ann Surg Oncol. 2006;13:1261–8. 16947009 10.1245/s10434-006-9023-y

[53] Margonis GA , Sergentanis TN , Ntanasis‐Stathopoulos I , et al. Impact of surgical margin width on recurrence and overall survival following R0 hepatic resection of colorectal metastases: a systematic review and meta‐analysis. Ann Surg. 2018;267:1047–55. 29189379 10.1097/SLA.0000000000002552

[54] Regimbeau JM , Cosse C , Kaiser G , et al. Feasibility, safety and efficacy of two‐stage hepatectomy for bilobar liver metastases of colorectal cancer: a LiverMetSurvey analysis. HPB (Oxford). 2017;19:396–405. 28343889 10.1016/j.hpb.2017.01.008

[55] Muratore A , Zimmitti G , Ribero D , Mellano A , Vigano L , Capussotti L . Chemotherapy between the first and second stages of a two‐stage hepatectomy for colorectal liver metastases: should we routinely recommend it? Ann Surg Oncol. 2012;19:1310–5. 21947627 10.1245/s10434-011-2069-5

[56] Vigano L , Torzilli G , Cimino M , et al. Drop‐out between the two liver resections of two‐stage hepatectomy. Patient selection or loss of chance? Eur J Surg Oncol. 2016;42:1385–93 27316601 10.1016/j.ejso.2016.03.020

[57] Sandstrom P , Rosok BI , Sparrelid E , et al. ALPPS improves resectability compared with conventional two‐stage hepatectomy in patients with advanced colorectal liver metastasis: results from a Scandinavian multicenter randomized controlled trial (LIGRO trial). Ann Surg. 2018;267:833–40. 28902669 10.1097/SLA.0000000000002511 PMC5916470

[58] Adam R , Imai K , Castro Benitez C , et al. Outcome after associating liver partition and portal vein ligation for staged hepatectomy and conventional two‐stage hepatectomy for colorectal liver metastases. Br J Surg. 2016;103:1521–9. 27517369 10.1002/bjs.10256

[59] Muhlbacher F , Huk I , Steininger R , et al. Is orthotopic liver transplantation a feasible treatment for secondary cancer of the liver? Transplant Proc. 1991;23:1567–8. 1989293

[60] Foss A , Adam R , Dueland S . Liver transplantation for colorectal liver metastases: revisiting the concept. Transpl Int. 2010;23:679–85. 20477993 10.1111/j.1432-2277.2010.01097.x

[61] Moris D , Tsilimigras DI , Chakedis J , et al. Liver transplantation for unresectable colorectal liver metastases: A systematic review. J Surg Oncol. 2017;116:288–97. 28513862 10.1002/jso.24671

[62] Dueland S , Guren TK , Hagness M , et al. Chemotherapy or liver transplantation for nonresectable liver metastases from colorectal cancer? Ann Surg. 2015;261:956–60. 24950280 10.1097/SLA.0000000000000786

[63] Hagness M , Foss A , Line PD , et al. Liver transplantation for nonresectable liver metastases from colorectal cancer. Ann Surg. 2013;257:800–6. 23360920 10.1097/SLA.0b013e3182823957

[64] Oxenberg J , Papenfuss W , Esemuede I , et al. Multidisciplinary cancer conferences for gastrointestinal malignancies result in measureable treatment changes: a prospective study of 149 consecutive patients. Ann Surg Oncol. 2015;22:1533–9. 25323473 10.1245/s10434-014-4163-y PMC4784677

[65] Jones RP , Vauthey JN , Adam R , et al. Effect of specialist decision‐making on treatment strategies for colorectal liver metastases. Br J Surg. 2012;99:1263–9. 22864887 10.1002/bjs.8835

[66] Morris EJ , Forman D , Thomas JD , et al. Surgical management and outcomes of colorectal cancer liver metastases. Br J Surg. 2010;97:1110–8. 20632280 10.1002/bjs.7032

[67] Neumann U , Denecke T , Pratschke J , et al. Evaluation for surgical treatment options in metastatic colorectal cancer (mCRC) – a retrospective, central evaluation of FIRE‐3. Ann Oncol. 2016;27:468.

